# 解剖性肺切除术后持续漏气预测模型构建

**DOI:** 10.3779/j.issn.1009-3419.2017.12.06

**Published:** 2017-12-20

**Authors:** 显宁 吴, 世斌 徐, 立 柯, 军 范, 君 王, 明然 解, 贤亮 江, 美青 徐

**Affiliations:** 230001 合肥，中国科学技术大学附属第一医院（安徽省立医院）胸外科 Department of Thoracic Surgery, The First Affiliated Hospital of University of Science and Technology of China (Anhui Provincial Hospital), Hefei 230001, China

**Keywords:** 解剖性肺切除, 持续漏气, 预测模型, 独立预测因子, Anatomic lung resection, Prolonged air leak (PAL), Prediction model, Independent predictors

## Abstract

**背景与目的:**

解剖性肺切除术后持续漏气（prolonged air leak, PAL）是胸外科常见并发症，重在准确预测及时预防，但目前国内尚缺少有效的预测模型，本研究旨在建立解剖性肺切除术后PAL临床预测模型。

**方法:**

回顾分析2016年1月-2016年10月安徽医科大学附属省立医院胸外科解剖性肺切除术患者的临床资料和术后漏气情况，其中A组病例359例，通过对患者的年龄（岁）、性别、身体质量指数（body mass index, BMI）、吸烟史、肺功能指数、手术方式（开放或腔镜，肺段、肺叶或其他，如支气管袖式或血管袖式）、手术切除肺叶位置、肺部病灶性质和胸腔粘连情况进行单因素及多因素分析，寻找解剖性肺切除术后PAL的独立预测因子，并建立临床预测模型。随后利用不同时期、不同治疗组完成的112例解剖肺切除患者作为B组，用于验证本模型的诊断效能，并绘制受试者工作特征（receiver operating characteristic curve, ROC）曲线。

**结果:**

多因素*Logistic*回归分析筛选出BMI、性别、吸烟史、第一秒用力肺活量占用力肺活量的百分比（forced expiratory volume in one second, FEV_1_%）、胸腔粘连及是否上叶切除为解剖性肺切除患者术后PAL的独立预测因子。利用筛选出的预测因子建立的诊断模型ROC曲线下面积为0.886（95%CI: 0.835-0.937），最佳临界值*P*=0.299，对应的诊断敏感性为78.5%，特异性为93.2%。

**结论:**

本研究建立的预测模型能较准确的预测解剖性肺切除术后PAL的发生，对及时有效预防PAL发生有指导作用。

## Abstract (en)

**Background and objective:**

Prolonged air leak (PAL) after anatomic lung resection is a common and challenging complication in thoracic surgery. No available clinical prediction model of PAL has been established in China. The aim of this study was to construct a model to identify patients at increased risk of PAL by using preoperative factors exclusively.

**Methods:**

We retrospectively reviewed clinical data and PAL occurrence of patients after anatomic lung resection, in department of thoracic surgery, Anhui Provincial Hospital Affiliated to Anhui Medical University, from January 2016 to October 2016. 359 patients were in group A, clinical data including age, body mass index (BMI), gender, smoking history, surgical methods, pulmonary function index, pleural adhesion, pathologic diagnosis, side and site of resected lung were analyzed. By using univariate and multivariate analysis, we found the independent predictors of PAL after anatomic lung resection and subsequently established a clinical prediction model. Then, another 112 patients (group B), who underwent anatomic lung resection in different time by different team, were chosen to verify the accuracy of the prediction model. Receiver-operating characteristic (ROC) curve was constructed using the prediction model.

**Results:**

Multivariate *Logistic* regression analysis was used to identify six clinical characteristics [BMI, gender, smoking history, forced expiratory volume in one second to forced vital capacity ratio (FEV_1_%), pleural adhesion, site of resection] as independent predictors of PAL after anatomic lung resection. The area under the ROC curve for our model was 0.886 (95%CI: 0.835-0.937). The best predictive P value was 0.299 with sensitivity of 78.5% and specificity of 93.2%.

**Conclusion:**

Our prediction model could accurately identify occurrence risk of PAL in patients after anatomic lung resection, which might allow for more effective use of intraoperative prophylactic strategies.

肺部术后持续漏气（prolonged air leak, PAL）是胸外科常见的并发症，也是胸外科医师必须解决的临床问题之一，据文献^[1, 2]^报道在肺部手术后其发生率约为11%-25%。术后持续漏气会延长胸腔引流管留置时间及住院时间，增加患者胸腔感染的风险，严重影响患者术后恢复及生活质量，且增加住院费用，造成有限医疗资源被占用^[3]^。针对肺术后持续漏气的治疗手段较多，但效果不一，其问题可能在于肺持续漏气已经发生，治疗方法虽多但属于补救性措施。如果我们能提前预测哪些患者可能发生术后持续漏气，我们就可以做到术中超前干预及术后早期预防，并且预测患者术后恢复的相关问题，做到提前告知沟通，减少不必要的医患矛盾。虽然国外有PAL相关模型报道^[4, 5]^，但是纳入的患者人群与我国患者人群临床特征存在较大差异，例如身体质量指数（body mass index, BMI）、年龄构成、吸烟史等，不能很好应用于我国患者。基于该设想，本研究旨在构建解剖性肺切除术后持续漏气的预测模型。

## 1 材料与方法

### 1.1 研究对象

回顾性分析安徽医科大学附属省立医院胸外科2016年1月-2016年10月期间因肺部疾病接受解剖性肺切除术患者的临床资料，其中2016年1月-2016年8月期间359例患者由甲治疗组完成解剖性肺切除术，该组样本定义为A组，作为建立*Logistic*回归模型的训练样本。2016年8月-2016年10月期间112例患者由乙治疗组完成解剖性肺切除术，该组样本定义为B组，作为*Logistic*回归模型的验证样本。

### 1.2 临床治疗流程

所有患者均因诊断肺部疾病需行解剖性肺切除，术前评估无绝对手术禁忌症，术中支气管及发育不全叶裂常规采用切割闭合器离断，肺部恶性病变依据美国国立综合癌症网络（National Comprehensive Cancer Network, NCCN）指南推荐行淋巴结采样或系统性淋巴结清扫，完成解剖性肺切除后采用试水膨肺法检测有无明显漏气，明显漏气处进行缝合修补，术中均不使用生物胶水及止血纱布等材料。手术结束患者于麻醉科复苏室拔除气管插管并转回胸外科普通病房，术后胸腔引流管自然引流，患者进行常规呼吸功能锻炼。患者胸腔引流管漏气情况每天评估2次，由责任医生早晚查房时完成并记录，PAL定义为患者接受解剖性肺切除术后胸腔引流管漏气时间超过7 d^[1, 6]^。胸引管拔除指征为：无漏气，24 h引流量少于200 mL，影像学检查提示肺复张满意。

### 1.3 排除标准

非解剖性肺切除患者：如楔形切除、瘤体剥除等；全肺切除患者；手术合并胸壁及膈肌切除重建；术后需机械通气；术后漏气确诊为支气管胸膜瘘；住院期间患者死亡；临床资料不全。

### 1.4 临床资料及处理

本研究收集的患者临床资料包括患者的年龄（岁）、性别、BMI、吸烟史、肺功能指数、手术方式（开放或腔镜，肺段、肺叶或其他，如支气管袖式或血管袖式）、手术切除肺叶位置、肺部病灶性质、胸腔粘连情况。本研究中我们对收集临床资料做以下处理：①BMI分为：＜19（消瘦）、19-24（正常）、＞24（肥胖）；②患者吸烟史由患者提供，由于具体包数难以精确统计，且既往研究发现吸烟包数与术后漏气并不相关^[7]^，在本研究中将其简化为无吸烟史和目前吸烟或既往有吸烟史两种情况；③肺功能指标选取第一秒用力肺活量占用力肺活量的百分比（forced expiratory volume in one second to forced vital capacity ratio, FEV_1_%）和一氧化碳弥散百分比（percentage of predicted diffusing capacity of the lung for carbon monoxide, DLco%），FEV_1_%和DLco%≥80%为正常，＜80%定义为异常；④根据术中粘连面积将胸腔粘连分为两种情况：无粘连或粘连面积局限于单个肺叶或粘连面积累计少于肺表面积的30%；粘连面积超过单个肺叶或累计粘连面积达到肺表面积30%及以上。

### 1.5 统计学方法

所有数据用SPSS 20.0统计软件进行处理：①单因素分析：符合正态分布、方差齐性的计量资料用均数±标准差表示，组间比较采用独立样本*t*检验；计数资料用频数和百分比表示，组间比较采用*χ*^2^检验。②多因素分析:对于单因素分析有统计学意义的因素，采用二元非条件*Logistic*回归分析筛选出解剖性肺切除术后持续漏气的独立预测因子，建立预测术后持续漏气的数学诊断模型。③将验证样本患者临床资料输入预测模型，计算出PAL预测发病率，并结合实际PAL发生情况，输入SPSS软件，分别计算出所有预测截点的敏感性、特异性和假阳性率（1-特异性），以敏感性为纵坐标代表真阳性率，（1-特异性）为横坐标代表假阳性率，绘制应用受试者工作特征曲线（receiver operating characteristic curve, ROC），统计软件输出曲线下面积（area under curve, AUC），AUC值在1.0和0.5之间。在AUC＞0.5的情况下，AUC越接近于1，说明诊断效果越好。AUC在0.5-0.7时有较低准确性，AUC在0.7-0.9时有一定准确性，AUC在0.9以上时有较高准确性。计算所有预测截点对应的约登指数（灵敏度+特异度-1），约登指数表示该预测截点发现真正阳性与假阳性的总能力；并结合ROC曲线，明确最佳临界值（cut-off值），标记对应的敏感性、特异性。*P*＜0.05为差异有统计学意义。

## 2 结果

### 2.1 构建模型的训练样本与验证样本患者一般资料比较

本研究调查的A组病例中，359例患者均接受解剖性肺切除，51例患者发生术后持续漏气，占14.2%。在B组112例患者中，18例患者发生术后持续漏气，占16.1%。为了明确验证样本是否适合用于模型的验证以及了解训练样本和验证样本的可比性，我们将A组及B组患者资料进行比较，验证样本在肺段切除及胸膜粘连患者占比上稍低于训练样本，而在吸烟、肺叶切除及恶性病变患者占比上稍高于训练样本，总体来说，两组患者异质性较小，具有可比性，具体如表 1所示。

**1:** A组和B组患者临床资料对比 Comparison of clinical data between group A and group B

Variable	Group A (*n*=359)	Group B (*n*=112)
Age (yr)	61.1±9.2	62.3±10.4
BMI (kg/m^2^)	24.5±5.2	24.8±4.3
Gender (male, *n*, %)	223 (62.1%)	65 (58.0%)
Smoker	181 (50.4%)	71 (63.4%)
VATS	309 (86.1%)	95 (84.8%)
Segementectomy	105 (29.2%)	20 (17.9%)
Lobectomy	238 (66.3%)	96 (85.7%)
FEV_1_%	81.7±11.5	85.0±9.7
DLco%	82.0±17.4	80.7±14.5
Pleural adhesion	91 (25.3%)	17 (15.2%)
Pathologic diagnosis (malignant)	292 (81.3%)	102 (91.1%)
Side of resection (right)	186 (51.8%)	55 (49.1%)
Site of resection (upper)	203 (56.5%)	59 (52.7%)
Air leak＞7 d	51 (14.2%)	18 (16.1%)
BMI: body mass index; FEV_1_%: forced expiratory volume in one second to forced vital capacity ratio; DLco: predicted diffusing capacity of the lung for carbon monoxide; VATS: video-assisted thoracic surgery.		

### 2.2 解剖性肺切除术后PAL单因素和多因素分析

单因素分析结果显示，解剖性肺切除术后发生PAL和无PAL患者在BMI、性别、吸烟史、FEV_1_%、胸腔粘连及是否上叶切除方面存在差异，具有统计学意义（*P*＜0.05）（表 2）。将单因素分析结果中具有统计学意义的因素作为自变量，通过二分类非条件*Logistic*回归进行分析，结果显示BMI、性别、吸烟史、FEV_1_%、胸腔粘连及是否上叶切除为解剖性肺切除患者术后PAL的独立预测因素（表 3）。

**2:** A组解剖性肺切除患者术后PAL的单因素分析 Univariate analysis of PAL after anatomic lung resection in group A

Variable		Air leak≤7 d (*n*=308)	Air leak＞7 d (*n*=51)	*t*/*χ*^2^	*P*
Age (yr)		60.8±10.9	61.4±11.0	0.298	0.766
BMI (kg/m^2^)		25.1±4.5	21.7±3.7	5.763	0.017
Gender	Male	181 (58.8%)	42 (82.4%)	10.344	0.001
	Female	127 (41.2%)	9 (17.6%)		
Smoking history	Yes	145 (47.1%)	36 (70.6%)	9.675	0.002
	No	163 (52.9%)	15 (19.4%)		
Surgical method 1	VATS	266 (86.4%)	43 (84.3%)	0.153	0.695
	Open	42 (13.6%)	8 (15.7%)		
Surgical method 2	Segementectomy	87 (28.2%)	18 (35.3%)	1.054	0.591
	Lobectomy	207 (67.2%)	31 (60.8%)		
	Other	14 (4.5%)	2 (3.9%)		
FEV_1_%	≥80%	187 (60.7%)	18 (35.3%)	11.542	0.001
	＜80%	121 (39.3%)	33 (64.7%)		
DLco%	≥80%	152 (49.4%)	21 (41.2%)	1.171	0.279
	＜80%	156 (50.6%)	30 (58.8%)		
Pleural adhesion	Yes	68 (22.1%)	23 (45.1%)	12.253	0.000
	No	240 (77.9%)	28 (54.9%)		
Pathologic diagnosis	Malignant	251 (81.5%)	41 (80.4%)	0.035	0.852
	Benign	57 (18.5%)	10 (19.6%)		
Side of resection	Right	159 (51.6%)	27 (52.9%)	0.03	0.862
	Left	149 (48.4%)	24 (47.1%)		
Site of resection	Upper	167 (54.2%)	36 (70.6%)	4.77	0.029
	None-upper	141 (45.8%)	15 (19.4%)		

**3:** 解剖性肺切除患者术后持续漏气的多因素*Logistic*回归分析结果 Results of multivariate *Logistic* regression analysis for patients with PAL after anatomic lung resection

Factor	B	S.E	Wals	*P*	OR	95%CI
BMI (kg/m^2^)	-0.688	0.284	5.859	0.015	0.503	0.288-0.877
Gender	1.037	0.398	6.785	0.009	2.820	1.293-6.153
Smoking history	1.026	0.480	4.562	0.033	2.790	1.088-7.152
FEV_1_%	1.878	0.454	17.105	＜0.001	6.539	2.686-15.922
Pleural adhesion	2.555	0.434	34.623	＜0.001	12.869	5.495-30.137
Site of resection	0.861	0.418	4.242	0.039	2.366	1.043-5.370
Constant	-4.194	0.743	31.828			
B: regression coefficient; S.E: standard error; OR: odds ratio; CI: confidence interval.						

### 2.3 *Logistic*回归模型的建立

解剖性肺切除术后PAL的预测模型: *P*=ex/（1+ex），X=-4.194-（0.688×BMI）+（1.037×性别）+（1.026×吸烟史）+（1.878×FEV_1_%）+（2.555×胸腔粘连）+（0.861×上叶切除）。公式中e为自然对数，BMI根据＜19（消瘦）、19-24（正常）、＞24（肥胖）分别赋值为1、2、3；若患者性别为男，有吸烟史，FEV_1_%＜80%，粘连面积超过单个肺叶或累计粘连面积达到肺表面积30%及以上，上叶肺切除则用1表示，否则为0。

### 2.4 模型验证及ROC曲线绘制

将B组112例患者临床资料代入本模型，计算B组患者每例患者行解剖肺切除术后发生PAL的几率，与最终临床观察是否发生PAL的结果进行对比，利用SPSS软件绘制ROC曲线（图 1）。ROC曲线下面积为0.886（95%CI: 0.835-0.937），最佳临界值*P*=0.299，对应的诊断敏感性为78.5%，特异性为93.2%。

**1:**
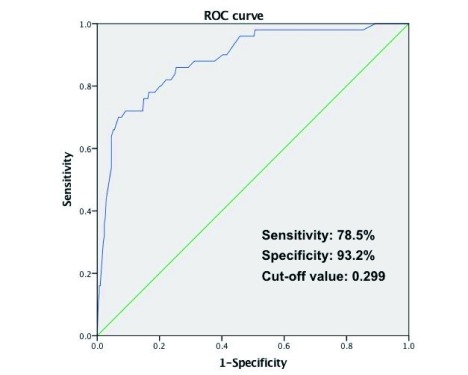
本模型ROC曲线下面积为0.886（95%CI: 0.835-0.937），最佳临界值*P*=0.299，对应的诊断敏感性为78.5%，特异性为93.2%。 The area under the ROC curve for our model was 0.886 (95%CI: 0.835-0.937). The best predictive *P* value was 0.299 with sensitivity of 78.5% and specificity of 93.2%.

## 3 讨论

PAL是肺切除术后常见的并发症，患者术后需长期带管，一方面会引起胸闷、疼痛，增加胸腔感染几率，并可能导致患者出现焦虑；另一方面会延长患者住院时间及住院费用^[8, 9]^。为了减少肺漏气的发生，胸外科医生不仅要在操作时小心谨慎，避免肺实质的撕裂；还要对明显可见的漏气进行仔细修补。然而术中未见明显漏气并不意味这术后持续漏气不会发生。

胸外科医生在预防肺术后漏气方面做了很大努力，不断对手术技术进行改进和创新，包括胸膜覆盖修补漏气、胸膜固定术以及切割缝合器配合可吸收材料垫片使用等^[10]^，这些技术取得了一定的效果，但存在一些争议及局限性，如常规做胸膜固定及胸膜修补漏气是否必要，过多材料的使用会增加费用等^[11]^。在术中应用新型的生物胶制剂变得越来越流行，一些单中心的研究结果显示术中使用生物胶，术后持续漏气发生率及胸引管留置时间确实减少了，但并未报道住院时间的缩短。一项基于Cochrane数据库的荟萃分析研究结果也显示，7项单中心研究中仅2项研究报道术后持续漏气发生率减低，14项研究中仅3项研究报道了住院日缩短；基于该结果作者认为不应推荐术中常规使用生物胶来预防术后持续漏气^[12]^。通过以上学习我们知道，在预防肺术后持续漏气工作中，我们不能所有患者一概而论，应该对术后持续漏气高危患者采用新的技术及生物胶制剂，这样才能做到有的放矢，提高实际效率。

本研究通过构建肺术后PAL的统计学模型，期望通过该模型来预测术后PAL发生的可能性。本研究选择接受解剖性肺切除术后的患者作为研究对象，包括了肺段切除和肺叶切除两种术式，主要考虑到以下几点：肺楔形切除术后持续漏气发生率较低，研究表明肺叶切除术后持续漏气发生率较高^[13]^，但肺段切除术后持续漏气的问题尚没有被重点讨论；近年来我国肺外科疾病谱正发生改变，越来越多的肺部小结节被诊断出来，肺段切除的术式成为胸外科热点。然而研究中我们通过单因素分析发现肺段与肺叶术式选择的不同与术后PAL发生无关，这与肺段切除的比例较低可能有关，还需要进一步研究。

通过单因素和多因素分析我们发现肺术后漏气与患者BMI、性别、吸烟史、FEV_1_%、胸腔粘连及是否上叶切除密切相关。究其根本，肺术后PAL关键因素有两类：存在肺损伤和影响损伤闭合相关因素。胸腔粘连与PAL发生密切相关基本得到公认^[1]^，并且在预测模型中所占权重最高也进一步证实该结论。主要原因是由于游离胸腔粘连大大增加了肺表面损伤的几率，而PAL的关键也在于肺表面胸膜的损伤、撕裂，即使是进行仔细的修补，也无法完全避免针眼漏气或遗漏微小破损的可能。

影响肺部破损闭合因素较多。本研究发现BMI低的患者更容易发生术后PAL，这可能与体型偏瘦患者的膈肌水平较低，胸腔瘦长，肺切除术后残肺即使复张完全，周围仍存在局部残腔，肺表面脏层胸膜与壁层胸膜不能很好贴合封闭肺表面破损。相对而言，BMI高的肥胖患者，因膈肌上抬，胸腔体积较小，肺复张后基本无残腔存在，即使有小的漏气也能很快闭合，从而减少PAL发生率。上叶切除患者也存在胸顶残腔无法及时闭合的问题，这可能是上叶患者术后PAL发生率更高的原因。肺功能检测中FEV1%是反映患者通气功能的指标，FEV_1_%也是诊断慢性阻塞性肺疾病的关键指标，FEV_1_%异常意味着患者的气道阻力增加，肺顺应性下降和肺实质气肿^[14]^；而这些又可能导致患者术后肺复张不全，且实质弹性较差不能很好地关闭肺表面小的破口，最终引起持续漏气，既往研究结果也表明FEV_1_%下降与PAL发生高度相关^[1, 15]^。我们还发现男性及有吸烟史患者PAL发生几率更高，这二者存在一定联系，我国男性吸烟比例明显高于女性，并且吸烟患者较吸烟人群肺气肿患病率更高，肺功能也更差，并且主要体现在通气功能障碍方面。

DLco%评估的是肺泡气体交换的功能，DLco%异常与肺术后低氧血症、呼吸衰竭等发生相关^[16]^，但本研究中并未发现其与PAL发生相关，我们也注意到纳入研究患者中弥散功能大多处于正常或轻度异常的水平，术前检查如明确为间质性肺病或换气功能明显异常，我们通常采取保留实质的楔形切除术或选用非手术治疗方案。有研究表明年龄大于65岁是肺术后PAL的影响因素^[11]^，本研究中发现年龄与解剖性肺切除术后PAL发生不相关，可能与我们纳入研究的A组、B组患者平均年龄较低有关，A组平均年龄（61.1±9.2）岁，B组平均年龄（62.3±10.4）岁，另一方面也说明我们的患者更加年轻化。

本研究中用于构建模型的训练样本与验证样本患者解剖性肺切除术后肺持续漏气发生率分别为14.2%、16.1%，与文献报道基本相符。我们构建模型的病例与验证病例来自不同治疗组、不同时期，验证结果显示诊断准确率较高，说明本研究建立的模型适用性较好。该模型最佳临界值*P*=0.299，对应的PAL诊断敏感性为0.785，特异性为0.932。因此根据该模型，我们对肺漏气发生概率超过29.9%的患者，我们术中需要仔细修补或应用新型的生物胶材料，以预防术后PAL的发生。

本研究的局限性在于模型的建立是基于单中心数据回顾性研究，将来需要多中心更大样本量的进一步深入研究。模型不够简化，临床医师使用起来较复杂，尚不能完全代替临床医生的经验性判断。在后期的工作中应联合多中心，扩大样本量，简化预测模型或构建出简易的评分系统；通过该预测模型我们可以预测患者肺术后发生持续漏气的可能性，并根据预测结果对肺漏气高风险患者在术中即采取干预措施，对低风险患者可以避免术中过度的操作及相关材料的使用。
